# Combining ability and heritability analysis of sweetpotato weevil resistance, root yield, and dry matter content in sweetpotato

**DOI:** 10.3389/fpls.2022.956936

**Published:** 2022-09-07

**Authors:** Immaculate Mugisa, Jeninah Karungi, Paul Musana, Roy Odama, Agnes Alajo, Doreen M. Chelangat, Milton O. Anyanga, Bonny M. Oloka, Iara Gonçalves dos Santos, Herbert Talwana, Mildred Ochwo-Ssemakula, Richard Edema, Paul Gibson, Reuben Ssali, Hugo Campos, Bode A. Olukolu, Guilherme da Silva Pereira, Craig Yencho, Benard Yada

**Affiliations:** ^1^National Crops Resources Research Institute (NaCRRI), NARO, Kampala, Uganda; ^2^Department of Agricultural Production, Makerere University, Kampala, Uganda; ^3^Department of Agronomy, Federal University of Viçosa, Viçosa, Minas Gerais, Brazil; ^4^International Potato Center, Kampala, Uganda; ^5^International Potato Center, Lima, Peru; ^6^Department of Entomology and Plant Pathology, University of Tennessee, Knoxville, TN, United States; ^7^Department of Horticultural Science, North Carolina State University, Raleigh, NC, United States

**Keywords:** clones, crosses, families, mixed models, *Cylas* spp., *Ipomoea batatas*

## Abstract

Efficient breeding and selection of superior genotypes requires a comprehensive understanding of the genetics of traits. This study was aimed at establishing the general combining ability (GCA), specific combining ability (SCA), and heritability of sweetpotato weevil (*Cylas* spp.) resistance, storage root yield, and dry matter content in a sweetpotato multi-parental breeding population. A population of 1,896 F_1_ clones obtained from an 8 × 8 North Carolina II design cross was evaluated with its parents in the field at two sweetpotato weevil hotspots in Uganda, using an augmented row-column design. Clone roots were further evaluated in three rounds of a no-choice feeding laboratory bioassay. Significant GCA effects for parents and SCA effects for families were observed for most traits and all variance components were highly significant (*p* ≤ 0.001). Narrow-sense heritability estimates for weevil severity, storage root yield, and dry matter content were 0.35, 0.36, and 0.45, respectively. Parental genotypes with superior GCA for weevil resistance included “Mugande,” NASPOT 5, “Dimbuka-bukulula,” and “Wagabolige.” On the other hand, families that displayed the highest levels of resistance to weevils included “Wagabolige” × NASPOT 10 O, NASPOT 5 × “Dimbuka-bukulula,” “Mugande” × “Dimbuka-bukulula,” and NASPOT 11 × NASPOT 7. The moderate levels of narrow-sense heritability observed for the traits, coupled with the significant GCA and SCA effects, suggest that there is potential for their improvement through conventional breeding *via* hybridization and progeny selection and advancement. Although selection for weevil resistance may, to some extent, be challenging for breeders, efforts could be boosted through applying genomics-assisted breeding. Superior parents and families identified through this study could be deployed in further research involving the genetic improvement of these traits.

## Introduction

Sweetpotato [*Ipomoea batatas* (L.) Lam] is the second most important root crop in sub-Saharan Africa (SSA) after cassava (FAOSTAT, 2020). The region is estimated to produce 28 million metric tons of sweetpotato annually, which translates to about 31% of the world’s total production for this crop (FAOSTAT, 2020). Its key attributes include its hardiness, fairly good performance under poor soil and weather conditions, and adaptation to a wide range of agro-ecological zones (Karyeija et al., 1998; Grüneberg et al., 2015). Additionally, orange-fleshed types possess nutritionally high pro-vitamin A content (Mwanga et al., 2009). Sweetpotato can be used as both human food and animal feed, making it one of the region’s most extensively produced root crops (Fuglie, 2007; Grüneberg et al., 2015; Gurmu et al., 2018). However, average yields of sweetpotato remain low in Africa, with estimates of about 6.8 metric t ha^−1^, compared to 20.8 metric t ha^−1^ in Asia and 24.5 metric t ha^−1^ in the United States (FAOSTAT, 2020). Several negative factors have contributed to this predicament such as pests and diseases that affect the crop, notably the African sweetpotato weevils, *Cylas puncticollis* (Boheman) and *Cylas brunneus* (Fabricius).

*Cylas* spp. are the most destructive pests of sweetpotato in SSA and are known to cause losses ranging from 60 to 100%; particularly during dry spells, and under heavy infestations (Chalfant et al., 1990; Smit, 1997; Capinera, 2006; Sorensen, 2009). Development and oviposition rates of these pests are temperature dependent, with optimal survival ranging between 25 and 30°C (Okonya et al., 2016). At 30°C, *Cylas* spp. require around 28 days to mature from egg to adult, and adults can live for another 94–309 days. Females lay between 94 and 350 eggs in their lifetime, with *C. puncticollis* females depositing 17 eggs per day on average (Musana et al., 2016; Okonya et al., 2016). The two African *Cylas* spp. usually occur together, co-infest the same plant, and develop within the same root, inflicting damage to storage roots and vines by tunneling into them (Anyanga et al., 2017). As a defense response, the plants release bitter-tasting and foul-smelling phenols and terpenes, further degrading root quality and rendering them unfit for either human or animal consumption (Chalfant et al., 1990; Ames et al., 1996).

Several cultural practices have been put forward for the management of sweetpotato weevils (SPWs) including using clean planting materials, crop rotation, intercropping, early planting and harvesting, hilling up, and removing alternate hosts (Talekar, 1992; Smit and Matengo, 1995; Smit, 1997). Chemical control using foliar fertilizers and dipping planting material in pesticides before planting has also been previously recommended (Allard, 1990; Hwang, 2000; Stathers et al., 2005). However, the effectiveness of these SPW management strategies is often variable because of the obscure feeding habits of the immature larvae that cannot be easily reached once inside the roots (Sorensen, 2009). Furthermore, the costs and limited access to insecticides in SSA further casts doubt on the feasibility and sustainability of this control method (Bassey, 2012). Okonya et al. (2014) reported that most sweetpotato growers do not take any steps to manage *Cylas* spp. from spreading in their gardens. Host plant resistance enhanced through genetic improvement, therefore, presents a major component for the integrated pest management (IPM) of these pests (Stathers et al., 2003; Stevenson et al., 2009; Anyanga et al., 2013), and breeding for resistance to *Cylas* spp. remains one of the key objectives in sweetpotato breeding programs in SSA.

The unknown sources of genetic resistance to weevils were formerly a major stumbling block in breeding (Mwanga et al., 2009; Stevenson et al., 2009; Anyanga et al., 2013). Over the past decade, however, researchers have identified genotypes, including landraces, with higher levels of resistance to weevils and with a potential for exploitation in breeding for resistance to these pests (Muyinza et al., 2012; Anyanga et al., 2013; Kagimbo et al., 2019). For instance, “New Kawogo,” a Ugandan landrace, has been shown to have moderate field resistance to *C. puncticollis* and *C. brunneus,* owing to the high concentration of hydroxycinnamic acid (HCA) esters on its root surfaces (Muyinza et al., 2012; Anyanga et al., 2013). “New Kawogo” is currently being utilized as parental material in breeding for SPW resistance in Uganda (Yada et al., 2015; Zhou, 2020) and in studying resistance mechanisms to *Cylas* spp. (Anyanga et al., 2017).

Another deterrent factor in sweetpotato weevil management particularly relating to molecular breeding for resistance to SPWs is the limited genomic resources for the crop. In the past, Yada et al. (2015) used 133 simple sequence repeat (SSR) markers to identify SPW resistance loci through regression analysis of a segregating population of 287 clones derived from a bi-parental cross between “New Kawogo,” and “Beauregard.” Twelve SSR markers were observed to be associated with SPW resistance (Yada et al., 2017a). More recently, Oloka (2019) also developed an integrated genetic linkage map of the same population using single nucleotide polymorphisms (SNPs) and identified four quantitative trait loci (QTL) associated with weevil resistance. Most of the markers and QTL published in these studies have hardly been utilized in crop improvement primarily because of the relatively low proportion of phenotypic variation explained by the QTL. Consequently, they had limited utility for marker assisted selection. However, efforts are currently underway to identify more QTL controlling SPW resistance using SNPs, in order to expedite genomic breeding for weevil resistance.

Nonetheless, there is currently a need for further identification and development of genotypes with more resistance to SPWs because the yield potential and storage root quality attributes of “New Kawogo” are not good and have been superseded by more recent varieties. Secondly, it is white-fleshed and lacks β-carotene (the precursor for vitamin A) that is found in the orange-fleshed varieties. There is presently a drive for vitamin A bio-fortified sweetpotato varieties, which are effective for combating vitamin A deficiency in Uganda and SSA at large (Yanggen and Nagujja, 2006). Furthermore, roots from this landrace have a shape that is not desired by the market. Therefore, there is a pressing need for further research and identification of superior genotypes in this regard.

To efficiently breed for resistance to SPWs, it is critical to have a comprehensive knowledge on the genetics of resistance to these pests and to identify parental genotypes that, when combined, result in superior progeny that outperform their parents in regard to weevil resistance and other key quality and agronomic traits such as storage root yield and dry matter content. Combining ability and heritability are key considerations when studying the genetics of crop traits (Ma Teresa et al., 1994). Combining ability is widely used by plant breeders in cultivar development to compare the performance of lines in hybrid combinations and to identify promising ones (Acquaah, 2007). It is a useful tool for identifying parents of superior genetic merit based on the performance of their offspring (Viana and Matta, 2003; Fasahat et al., 2016).

General combining ability (GCA) refers to the mean performance of a parental genotype in different cross combinations whereas specific combining ability (SCA) defines the performance of a specific cross combination regarding a particular trait and is based on the mean performance of the lines involved (Falconer and Mackay, 1996; Acquaah, 2007). Whereas GCA is ascribed to additive gene action, SCA is a result of non-additive genetic variance contributed by dominance, overdominance, or epistasis (Acquaah, 2007). Heritability, on the other hand, estimates the proportion of phenotypic variance that is due to genetic components (Bernardo, 2014). It indicates the extent to which a particular trait can be passed on from parents to their progeny. Narrow sense heritability, which represents the proportion of total phenotypic variance attributable to additive gene action, is important because the effectiveness of selection is dependent on the additive component of genetic variance relative to total variance (Acquaah, 2007; Bernardo, 2014).

Studies on the inheritance of sweetpotato traits are rather challenging because of its complex genome. Sweetpotato is a highly heterozygous autohexaploid (2*n* = 6*x* = 90) with complex segregation ratios, thus complicating studies on its genetics and cytology (Jones, 1986; Ukoskit and Thompson, 1997). Despite this, it is possible to partition genetic variation into components including general combining ability and specific combining ability (Mwanga et al., 2002) and as such, obtain a relative estimate of the inheritance of a given trait.

Mating designs that estimate GCA and SCA of quantitative traits are important in breeding heterozygous crops. The North Carolina (NC) II mating design is a factorial design that has previously been used by researchers to study inheritance in sweetpotato (Gasura et al., 2008; Sseruwu, 2012; Kagimbo et al., 2019). This design enables breeders to obtain information about combining ability, using less labour and resources compared to a full diallel (Comstock and Robinson, 1952; Acquaah, 2007). It is thus advantageous over the diallel method when many parents are involved (Hallauer and Miranda, 1988).

Estimates of heritability and combining ability are usually specific to the trait, population, and environments being tested (Acquaah, 2007). An understanding of the heritability of a trait in a given population can guide a breeder in choosing the most appropriate selection strategy and in making the right decisions that would maximize genetic gain. Analysis of combining ability further enables breeders to identify the most suitable combiners for hybridization, in addition to identifying crosses with exceptional performance. This underscores the need to study these parameters in the present population. This study was carried out to determine the general combining ability and specific combining ability, and to estimate the heritability of sweetpotato weevil (*Cylas* spp.) resistance, storage root yield, and dry matter content in a multi-parental sweetpotato population.

## Materials and methods

### Description of germplasm

A population of 1,896 F_1_ progeny, developed from an 8 × 8 paired crossing design, was used in this study. The population, known as the Mwanga Diversity Panel (MDP), was developed in Uganda in 2016 and 2017 at the National Crops Resources Research Institute (NaCRRI), Namulonge, under the Genomic Tools for Sweetpotato improvement project (GT4SP). The 16 parental lines that were crossed to develop this population were sourced from a hybrid breeding pool that comprised of two separate polycross breeding nurseries: population A (50 clones) and population B (80 clones; David et al., 2018). The progenitors originated from four different countries, with the majority from Uganda (Wu et al., 2018; Zhou, 2020).

Parental lines were selected based on desirable attributes for a sweetpotato ideotype including, high dry matter content and storage root yields, beta-carotene content (for the orange-fleshed types), and resistance to key biotic stresses, such as sweetpotato virus disease, Alternaria disease, and sweetpotato weevils (Table 1). The 16 parental lines were crossed (8B × 8A) using the NCII mating design, without reciprocals that resulted in 64 families. All eight pairs of crosses were successful, with over 100 seeds generated cross. The seeds were thereafter transferred to Biosciences eastern and central Africa (BecA) in Kenya, where they were germinated and raised *in vitro*. The resulting lines (approximately 30 clones per cross), were later returned to Uganda where the seedlings were initially raised in a screen house, and later on planted in the field.

**Table 1:** Description of parental material utilized in the 8 × 8 paired cross.

Code	Genotype	Response to *Cylas* spp.	Origin
Male parents			
A1	“Ejumula”	Susceptible	Uganda
A2	NASPOT 1	Susceptible	Uganda
A3	“Dimbuka-bukulula”	Susceptible	Uganda
A4	NASPOT 5/58	Susceptible	Uganda
A5	NASPOT 7	Susceptible	Uganda
A6	SPK004	Susceptible	Kenya
A7	NASPOT 10 O	Susceptible	Uganda
A8	NK259L	Moderately resistant	Uganda
Female parents			
B1	“Resisto”	Susceptible	USA
B2	“Magabali”	Moderately resistant	Uganda
B3	NASPOT 5	Moderately resistant	Uganda
B4	“Wagabolige”	Moderately resistant	Uganda
B5	“Mugande”	Susceptible	Uganda
B6	NASPOT 11	Susceptible	Uganda
B7	“New Kawogo”	Moderately resistant	Uganda
B8	“Huarmeyano”	Moderately resistant	Peru

### Study sites

Field experiments were conducted at two locations within Uganda, namely, Abi Zonal Agricultural Research and Development Institute (ZARDI), in Arua district; and Ngetta ZARDI, in Lira district (Table 2), respectively. The two sites were located in sweetpotato weevil hotspots in different agro-ecological zones of the country. Both field trial sites experience bi-modal rainfall patterns annually. In addition, a no-choice feeding bioassay was carried out at the sweetpotato entomology laboratory at the National Crops Resources Research Institute (NaCRRI) located in central Uganda.

**Table 2:** Description of field study sites.

Description	Field sites	
	Abi ZARDI	Ngetta ZARDI
Latitude	3°4′37.2” N	2°16′10.8”N
Longitude	30°56′34.6″E	32°53′57.2″E
Altitude (m.a.s.l)	1,211	1,080
Agro-ecological zone^a^	Northwestern wooded savannah	Northern moist farmlands
Soil type^b^	Sandy Clay Loam	Sandy Loam
Annual total rainfall (mm)	1,250	1,361
Mean annual temperature (°C)	24	23.4

m.a.s.l, meters above sea level.

a Wortmann and Eledu, 1999.

b Kaizzi et al., 2012.

### Trial design, establishment, and management

#### Field trials

Field trials were set up using an augmented row-column design with two replicated checks, one moderately resistant (“New Kawogo”) and the other susceptible (“Ejumula”) to sweetpotato weevils. The 1,896 test progeny, 16 parental genotypes, and two checks were randomized within 46 rows and 43 columns, and the checks were replicated in every column at each field site, making a total of 86 check plots per site and 344 checks in total. Field trials were conducted during the first and second rainy seasons of 2019 (2019A and 2019B, respectively) at Abi ZARDI, and in 2019B and 2020A (first rainy season of 2020) at Ngetta ZARDI. High quality, uniform sweetpotato vines of approximately 30 cm long were planted on ridges (3 m long and 60 cm high, knee length) at a spacing of 30 cm between plants and 1 m between ridges. Field management was implemented through regular weeding using hoes, three times during each season. Fertilizer and irrigation were not applied during these field experiments. Storage roots were harvested at 5 months after planting (unlike the 4 months when farmers usually start harvesting) in order to give time for weevil population buildup.

#### No-choice feeding laboratory bioassay

Clones that survived in the field and produced at least 3–5 clean, uninfested storage roots at harvest were further evaluated in the entomology laboratory at controlled room temperature (25 ± 2°C) in a no-choice feeding bioassay. A total of 1,360 clones were tested using a randomized complete block design (RCBD) with three replicates and two checks (same checks used for field trials). Each replicate was placed on its own shelf in the laboratory, with each of the three shelves serving as a block. Three healthy storage roots of approximately equal weight (±200 g) were selected at harvest from the field trial for each clone. The roots were carefully selected to ensure that they were free from any visibly noticeable weevil punctures or damage. These were cleaned, dried, and then placed in separate plastic jars (1.5-L capacity) such that each jar contained one storage root per clone. The roots were then artificially inoculated with 10 2-week-old gravid female adult weevils (*C. puncticollis*).

The weevils used in this study were obtained from a *C. puncticollis* mother culture that is usually maintained at the NaCRRI sweetpotato entomology laboratory on the clone NAROSPOT 1. From the NaCRRI mother culture, a subculture consisting of 20–30 males and females were placed in containers (2-liter capacity) to allow for mating. After a two-week period, 10 gravid female weevils were then introduced into jars and their tops covered using muslin cloth to allow aeration. They were allowed to feed and oviposit for 24 h, after which the weevils were removed from each jar and the number of feeding holes on the roots counted and recorded; following the methodologies of Anyanga et al. (2017). The eggs that were laid were left to incubate until emergence at room temperature (25 ± 2°C). A total of three runs/rounds of this trial were conducted with storage roots that had been harvested from Abi ZARDI in 2019A and Ngetta ZARDI in 2019B and 2020A.

### Data collection

For the field trials, data were collected on weevil severity (WED), weevil incidence (WI), and storage root yield (SRY) at harvest. Weevil severity was assessed by inspection of harvested storage roots in each plot and scoring using a scale of 1–9 as described by Grüneberg et al. (2019) where: 1 = no damage; 3 = minor; 5 = moderate; 7 = heavy; and 9 = severe damage, with numbers in between representing intermediate ratings. On the other hand, we determined weevil incidence by counting the number of infested and non-infested roots per plot after which we expressed the number of infested roots as a percentage of the total number of roots. At harvest, we separately weighed marketable (root diameter > 3 cm, having no cracks, insect damage, and/or rotten parts) and non-marketable roots (root diameter < 3 cm, with cracks, insect damage, and/or rotten parts). Total storage root yield was then obtained by adding the weight of marketable and non-marketable roots in each plot and expressing the total weight in tons per hectare.

After harvest, the percentage dry matter content (DMC) of each clone was obtained by slicing two clean randomly selected medium-sized (approximately 200–300 gm) fresh storage roots into small chips. A composite sub-sample of about 100–200 g was then obtained, placed in a well-labeled paper bag, and weighed to determine the exact fresh weight. The sub-sample was then dried at 70°C in an oven for 72 h until a constant mass was attained. The dry mass was thereafter weighed and the weight was expressed as a percentage of the fresh weight (Islam et al., 2002; Kagimbo et al., 2019). For the no-choice bioassay, data was collected on the number of feeding holes (FH) for each clone after roots had been exposed to weevils for 24 h as described by Anyanga et al. (2017). The number of adults that emerged from the roots was also counted on a weekly basis from day 25 to 67 after artificial infestation, after which the trial was terminated.

### Data analysis

Data analysis was done using the package ASReml-R v. 4.1 (Gilmour et al., 2015) in R statistics software, v. 4.0 (R Development Core Team, 2019). The combination of sites and seasons formed four field trial environments: two sites by two seasons each. A joint mixed-model for each trait was used as follows:


$$y=X\beta +Zg+Ti+e_{\xi \eta }$$

where y refers to the vector of phenotypic observations, β is the vector of fixed effects of environments, and X is the associated design matrix; g is the random vector of genotypic effects, $g\sim N\left(0,A\sigma_{g}^{2}\right)$ , where A is a pedigree-based relationship matrix among genotypes and $\sigma_{g}^{2}$ is the additive genetic variance, and *Z* is the associated design matrix; i is the random vector of genotype by environment interaction, $i\sim N\left(0,I\sigma_{i}^{2}\right)$ , where I is an identity matrix and $\sigma_{i}^{2}$ is the genotype by environment variance, and *T* is the associated design matrix; $e_{\xi \eta }$ is the random vector of residuals, modeled by using an auto-regressive order 1 (AR1 × AR1) process in the row and column directions within each environment. The pedigree-based relationship matrix ( A ) was computed based on Kerr et al. (2012) using the *Amatrix* function of the R package AGHmatrix v. 2.0.4 (Amadeu et al., 2016) considering autohexaploidy.

Variance components for weevil severity and incidence, storage root yield, and dry matter content were estimated by the Restricted Maximum Likelihood (REML) method and best linear unbiased predictions (BLUPs) were obtained. Variance components were then tested against the reduced model (without a specific random effect) using likelihood ratio tests (LRT). The narrow-sense heritability ( $h^{2})$ for each field trait across environments was estimated by the model without spatial variation, i.e., assuming $e\sim N\left(0,I\sigma_{e}^{2}\right)$ , according to Cullis et al. (2006) as follows:


$$h^{2}=1-\frac{{\bar{v}}_{\Delta }^{\mathrm{BLUP}}}{2\sigma_{g}^{2}}$$

where ${\bar{v}}_{\Delta }^{\mathrm{BLUP}}$ is the average standard error of the genotypic BLUPs and $\sigma_{g}^{2}$ is the additive genetic variance.

For the bioassay, the joint model used for each trait was as follows:


y=Xβ+Zg+Ti+Qr+e

where r is the random vector of replicate within environment, $r\sim N\left(0,I\sigma_{r}^{2}\right)$ , where $\sigma_{r}^{2}$ is the replicate within environment variance and Q is the associate design matrix, e is the random vector of independent residuals, $e\sim N\left(0,I\sigma_{e}^{2}\right)$ , where $\sigma_{e}^{2}$ is the residual variance.

The variance components for GCA effects for males and females, and SCA effects for crosses for each trait across environments were obtained using the following mixed model:


$$\begin{array}{l} y=X\beta +Z_{A}g_{A}+Z_{B}g_{B}+Z_{C}g_{C}+Z_{AE}g_{AE}\\ \;\;\;\;\;\;\;\;\;\;\;\;\;\;\;\;\;\;\;\;+Z_{BE}g_{BE}+Z_{CE}g_{CE}+e_{\xi \eta } \end{array}$$

where y refers to the vector of phenotypic observations, β is the vector of fixed effects of environments, and X is the associated design matrix; $g_{A},g_{B},g_{C}$ are random vectors of GCA for males, GCA for females, and SCA for crosses, respectively, with $g_{A}\sim N\left(0,I\sigma_{A}^{2}\right)$ , $g_{B}\sim N\left(0,I\sigma_{B}^{2}\right)$ , and $g_{C}\sim N\left(0,\;D\sigma_{C}^{2}\right)$ , where *D* is a matrix that reflects the number of parents shared by the different crosses (Kerr et al., 2012), computed using “1” for crosses that shared both parents, “½” for those that shared one parent, and “0” for crosses that did not share any parent; $\sigma_{A}^{2},\sigma_{B}^{2},\sigma_{C}^{2}$ are genetic variances of males, females, and crosses; $Z_{A},Z_{B},Z_{C}$ are the respective incidence matrices; $g_{AE},g_{BE},g_{CE}$ are random vectors of GCA for males by environment interaction, GCA for females by environment interaction, and SCA for crosses by environment interaction, respectively, with $g_{AE}\sim N\left(0,I\sigma_{AE}^{2}\right)$ , $g_{BE}\sim N\left(0,I\sigma_{BE}^{2}\right)$ , and $g_{CE}\sim N\left(0,I\sigma_{CE}^{2}\right)$ ; $\sigma_{AE}^{2},\sigma_{BE}^{2},\sigma_{CE}^{2}$ are genetic variances of males by environment interaction, females by environment interaction, and crosses by environment interaction, and $Z_{AE},Z_{BE},Z_{CE}$ are the respective incidence matrices. The *D* matrix was built based on the number of parents that a given pair of crosses may share. Considering families *i* and *j*, *D*_*ij*_ = 1 if families *i* and *j* share both parents, $D_{ij}=\frac{1}{2}$ if *i* and *j* share the one parent, and *D*_*ij*_ = 0 if *i* and *j* do not share any parent.

Pearson’s correlation analysis based on predicted mean values was conducted to observe the associations between weevil-related parameters recorded in the field and in the laboratory bioassay.

## Results

### Variance component parameters and heritability estimates

The REML analysis of traits measured in the field trials revealed that highly significant variance components (*p* ≤ 0.001) existed for genotype effects for weevil severity, incidence, storage root yield, and dry matter content. The variance components associated with genotype by environment (G × E) interactions were similarly highly significant (*p* ≤ 0.001) for the four traits (Table 3). Estimates of narrow-sense heritability ( $h^{2}$ ) for field traits were moderate (Table 3). Significant variation was recorded in GCA effects of female parents (GCAf) for weevil incidence (*p* ≤ 0.05) and storage root yield (*p* ≤ 0.01), GCAf by environment interaction for WED (*p* ≤ 0.05), and between families for SRY and DMC (*p* ≤ 0.05; Table 4). However, variance components associated with GCA effects of male parents (GCAm) were all non-significant. SCA variances for WED and WI were also non-significant (Table 4).

**Table 3:** Variance components, heritabilities ( $h^{2}$ ), and likelihood ratio test results for weevil severity (WED, scale 1–9), weevil incidence (WI, %), storage root yield (SRY, t/ha), and dry matter content (DMC, %) across four environments.

Variance components	Traits			
	*WED*	WI	SRY	DMC
Genotype	0.40^***^	11.94^***^	6.37^***^	7.97^***^
Genotype × Environment	0.91^***^	72.67^***^	6.17^***^	7.21^***^
Env_Abi2019A	3.66	743.70	13.12	34.30
Env_Abi2019B	2.60	708.90	26.37	17.71
Env_Ngetta2019B	2.11	616.31	11.55	83.26
Env_Ngetta2020A	1.89	162.46	29.31	192.50
				
*h* ^2^	0.35	<0.01	0.36	0.45

*** Significant at *p* ≤ 0.001. Residual variances presented separately for each of the four environments.

**Table 4:** Variance components and likelihood ratio test results for general combining ability (GCA) and specific combining ability (SCA) effects for weevil severity (WED, scale 1–9), weevil incidence (WI, %), storage root yield (SRY, t/ha), and dry matter content (DMC, %) across four environments.

Variance components	Traits			
	*WED*	WI	SRY	DMC
GCAf	0.02	4.68^*^	1.05^**^	0.53
GCAm	0.00	1.70	0.00	0.00
GCAf × Environment	0.02^*^	3.61	0.65^*^	0.36^**^
GCAm × Environment	0.00	0.47	0.24^*^	0.15
SCA	0.01	0.00	0.42^*^	0.54^*^
SCA × Environment	0.01	1.82	0.00	0.00
Env_Abi2019A	4.52	715.96	36.22	22.11
Env_Abi2019B	3.54	764.38	22.33	34.71
Env_Ngetta2019B	3.17	671.92	90.88	21.33
Env_Ngetta2020A	2.92	221.57	205.24	36.97

* Significant at *p* ≤ 0.05.

** Significant at *p* ≤ 0.01. Residual variances presented separately for each of the four environments.

Analysis of data obtained from the no-choice feeding bioassay showed significant variance components among genotypes (*p* ≤ 0.01) and G × E (*p* ≤ 0.05) for the number of adult weevils, *C. puncticollis* (Table 5). The case was different for the number of feeding holes, where significant variance components (*p* ≤ 0.01) were recorded for G × E interactions but not for genotypes (Table 5). We were unable to obtain BLUPS for genotypes for the number of feeding holes out of the model that was used for the analysis, so we limited our discussions to the other traits for which predictions were successfully obtained in the REML analysis (significant genetic variances).

**Table 5:** Variance components and likelihood ratio tests for number of emerged adult weevils (CP) and number of feeding holes (FH) tested for three rounds of the no-choice laboratory bioassay.

**Variance components**	**Traits**	
	***CP***	**FH**
Rep × Environment	290.53^***^	1.53^*^
Genotype	45.60^**^	2.33
Genotype × Environment	268.10^*^	24.40^**^
Residual	2971.13	439.11

* Significant at *p* ≤ 0.05.

** Significant at *p* ≤ 0.01.

*** Significant at *p* ≤ 0.001. Residual variances presented separately for each of the four environments.

**Figure 1:**
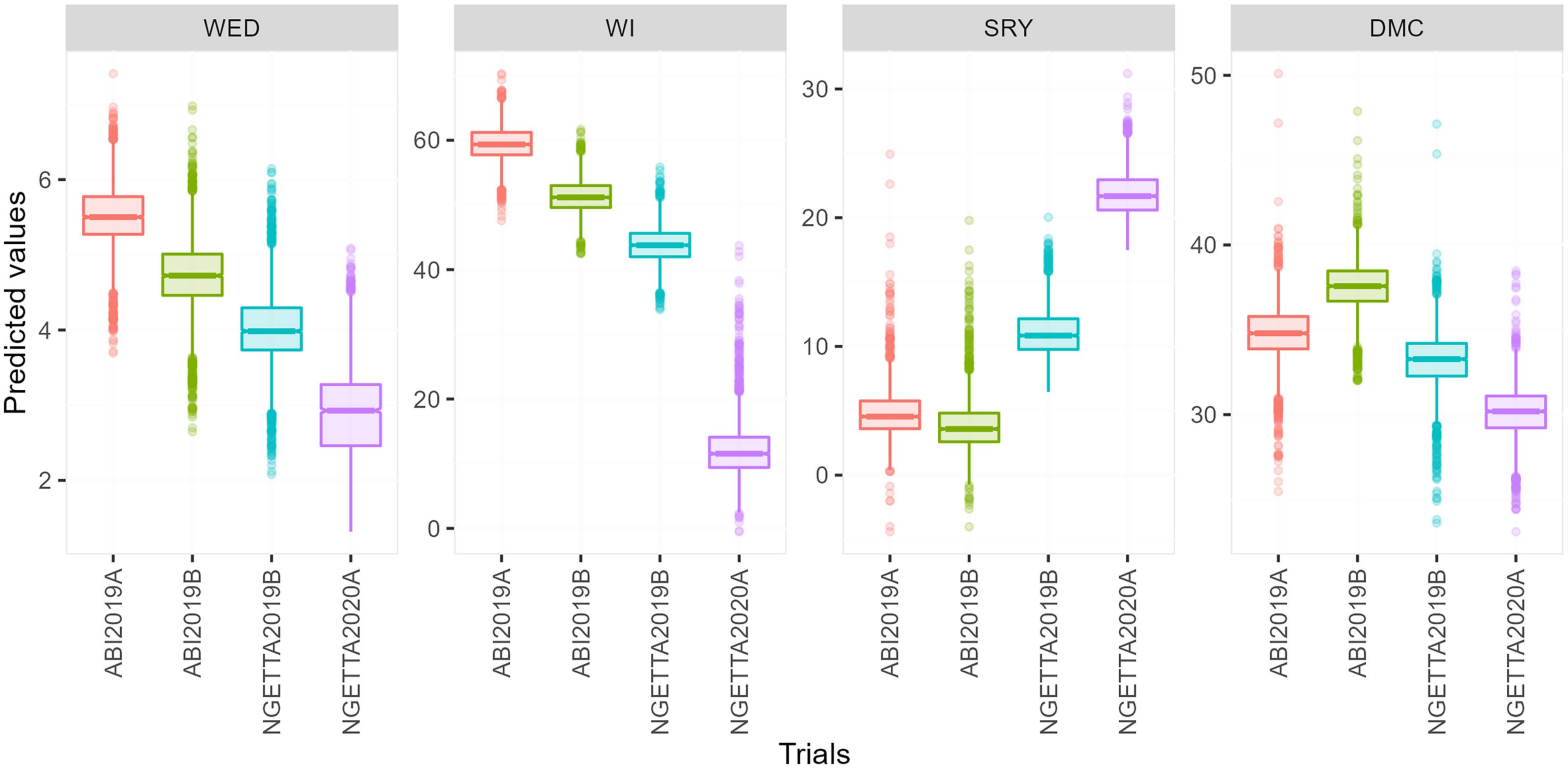
Boxplots of predicted means for weevil severity, weevil incidence, storage root yield, and dry matter content across four environments.

Among the four environments, the highest predicted means for weevil severity and weevil incidence were recorded in Abi ZARDI in 2019A, whereas the lowest were in Ngetta ZARDI in season 2020A. Conversely, the highest predicted means for storage root yield and lowest dry matter content were observed in Ngetta ZARDI in 2020A, while the lowest mean root yield and highest mean dry matter content were from Abi ZARDI in season 2019B (Figure 1).

Pearsons’ correlation analysis was done between weevil severity, incidence and the number of adult weevils recorded in the bioassay. A moderately strong, positive and highly significant (*r* = 0.51*; p* ≤ 0.001) relationship was observed between weevil severity and weevil incidence. However, a weak, negative and significant association (*r* = −0.31*; p* ≤ 0.01) existed between field weevil severity and the number of adult weevils that emerged from clones in the bioassay. Similarly, the correlation between weevil incidence and the number of adult weevils was also weak and negative, but not significant (*r =* −0.11*; p* = *0.2*).

### General and specific combining ability effects

The GCA and SCA effects for parental genotypes and the 64 families are presented in Tables 6, 7 respectively. GCA predictions of parental clones for WED scores ranged from 4.11 (“Mugande”) to 4.47 (NASPOT 11) whereas for WI ranged from 38.77% (“Mugande”) to 44.35% (NASPOT 11). “Ejumula,” the susceptible check, and Dimbuka-bukulula recorded the highest (43.26%) and smallest (40.03%) predictions for WI among the male parents across environments. GCA predictions for storage root yield ranged from 9.45 to 12.1 t ha^−1^ in parental genotypes, with the highest values in NASPOT 5 and “New Kawogo” and the lowest in NASPOT 5and “Huarmeyano.” GCA predictions for DMC were highest in NASPOT 11 (34.76%) and lowest in “Resisto” (32.41%). In the no-choice feeding bio-assay, NASPOT 5 (64.56) and “Ejumula” (60.24) recorded the highest GCA for number of adult weevils (CP), whereas “Wagabolige” (52.02) and Resisto (52.47) had the lowest values.

**Table 6:** General combining ability (GCA) predictions for weevil severity (WED, scale 1–9), weevil incidence (WI, %), storage root yield (SRY, t/ha), dry matter content (DMC, %), and number of emerged adult weevils (CP) for the 16 sweetpotato parental genotypes.

	**Trait**				
	**WED**	**WI**	**SRY**	**DMC**	^**1**^**CP**
**Female parents**
Huarmeyano	4.32	40.64	9.69	34.14	59.16
Magabali	4.27	42.34	11.31	34.31	55.69
Mugande	4.11	38.77	9.78	34.08	57.03
NASPOT 11	4.47	44.35	12.09	34.76	54.69
NASPOT 5	4.24	39.31	9.45	33.58	64.56
New Kawogo	4.41	44.17	12.04	34.28	56.34
Resisto	4.44	43.98	10.57	32.41	52.47
Wagabolige	4.26	40.64	10.14	34.22	52.02
**Male parents**					
Dimbuka-bukulula	4.30	40.03	10.66	33.98	57.25
Ejumula	4.31	43.26	10.62	34.06	60.24
NASPOT 1	4.31	42.61	10.71	33.94	55.93
NASPOT 10 O	4.30	41.97	10.76	34.00	54.97
NASPOT 7	4.30	40.86	10.86	33.96	57.15
NASPOT5/58	4.30	41.45	10.50	34.03	55.09
NK259L	4.30	42.23	10.81	33.95	53.93
SPK004	4.30	41.13	10.64	34.03	58.20

1 Results from the no-choice bioassay trial.

**Table 7:** Specific combining ability (SCA) predictions for weevil severity (WED, scale 1–9), weevil incidence (WI, %), storage root yield (SRY, t/ha), dry matter content (DMC, %), and number of emerged adult weevils (CP) for the 64 sweetpotato families.

**Family**	**Traits**				
	**WED**	**WI**	**SRY**	**DMC**	^**1**^**CP**
Resisto × Ejumula	4.39	41.64	10.20	33.91	56.76
Resisto × NASPOT 1	4.43	42.01	10.45	33.03	55.80
Resisto × Dimbuka-Bukulula	4.31	41.66	10.91	33.54	56.38
Resisto × NASPOT5/58	4.36	41.71	10.14	33.63	56.21
Resisto × NASPOT 7	4.35	41.59	11.41	33.74	57.19
Resisto × SPK004	4.30	41.58	10.45	33.83	56.06
Resisto × NASPOT 10 O	4.27	41.76	10.37	34.16	56.68
Resisto × NK259L	4.31	41.54	11.48	33.22	57.83
Magabali × Ejumula	4.40	41.98	10.81	34.63	56.73
Magabali × NASPOT 1	4.32	41.60	10.81	33.88	56.73
Magabali × Dimbuka-Bukulula	4.28	41.44	10.69	33.53	56.75
Magabali × NASPOT5/58	4.28	41.51	10.26	34.67	57.46
Magabali × NASPOT 7	4.27	41.63	11.26	33.51	55.51
Magabali × SPK004	4.22	41.45	10.60	34.53	55.85
Magabali × NASPOT 10 O	4.27	41.89	10.72	34.32	56.71
Magabali × NK259L	4.35	41.83	11.05	33.44	55.10
NASPOT 5 × Ejumula	4.31	41.34	9.99	33.87	57.12
NASPOT 5 × NASPOT 1	4.38	41.84	10.76	33.89	56.05
NASPOT 5 × Dimbuka-Bukulula	4.22	41.30	10.46	34.24	56.31
NASPOT 5 × NASPOT5/58	4.33	41.63	10.18	33.89	56.73
NASPOT 5 × NASPOT 7	4.33	41.75	10.86	33.78	56.47
NASPOT 5 × SPK004	4.23	41.59	10.28	33.46	57.22
NASPOT 5 × NASPOT 10 O	4.30	41.93	11.21	33.82	56.39
NASPOT 5 × NK259L	4.24	41.64	10.47	34.22	57.50
Wagabolige × Ejumula	4.40	41.76	10.86	34.41	57.06
Wagabolige × NASPOT 1	4.40	41.77	11.04	33.47	56.02
Wagabolige × Dimbuka-Bukulula	4.26	41.53	10.61	34.02	57.14
Wagabolige × NASPOT5/58	4.22	41.65	9.90	34.60	59.64
Wagabolige × NASPOT 7	4.30	41.65	10.29	34.04	57.22
Wagabolige × SPK004	4.22	41.64	10.81	34.22	56.89
Wagabolige × NASPOT 10 O	4.25	41.23	10.55	33.89	56.89
Wagabolige × NK259L	4.31	41.92	10.89	33.70	56.02
Mugande × Ejumula	4.36	41.88	10.26	34.87	55.86
Mugande × NASPOT 1	4.23	41.51	10.65	33.70	55.68
Mugande × Dimbuka-Bukulula	4.20	41.40	10.23	33.92	57.08
Mugande × NASPOT5/58	4.35	41.47	10.61	34.37	56.49
Mugande × NASPOT 7	4.28	41.67	11.01	33.69	56.91
Mugande × SPK004	4.26	41.72	10.47	34.46	57.61
Mugande × NASPOT 10 O	4.18	41.72	10.44	33.27	57.92
Mugande × NK259L	4.21	41.59	10.89	33.80	55.83
NASPOT 11 × Ejumula	4.49	42.10	10.75	34.68	56.26
NASPOT 11 × NASPOT 1	4.38	41.55	10.45	33.56	55.74
NASPOT 11 × Dimbuka-Bukulula	4.38	42.03	11.03	34.29	56.60
NASPOT 11 × NASPOT5/58	4.37	41.86	10.53	34.33	56.71
NASPOT 11 × NASPOT 7	4.24	41.40	11.22	34.17	56.85
NASPOT 11 × SPK004	4.26	41.52	11.08	34.50	55.56
NASPOT 11 × NASPOT 10 O	4.28	41.63	10.86	34.09	57.97
NASPOT 11 × NK259L	4.35	41.45	11.12	33.71	56.47
New Kawogo × Ejumula	4.38	41.36	10.19	34.33	56.58
New Kawogo × NASPOT 1	4.35	41.67	10.84	33.89	56.39
New Kawogo × Dimbuka-Bukulula	4.31	41.69	11.07	34.21	56.71
New Kawogo × NASPOT5/58	4.36	41.77	10.25	34.17	56.59
New Kawogo × NASPOT 7	4.28	41.42	11.59	33.82	56.18
New Kawogo × SPK004	4.30	41.94	10.87	34.19	56.32
New Kawogo × NASPOT 10 O	4.32	41.72	11.23	33.85	56.29
New Kawogo × NK259L	4.34	41.95	10.94	33.98	57.48
Huarmeyano × Ejumula	4.44	41.76	10.86	34.25	55.97
Huarmeyano × NASPOT 1	4.37	41.65	10.81	34.09	55.63
Huarmeyano × Dimbuka-Bukulula	4.27	41.63	9.87	33.64	56.56
Huarmeyano × NASPOT5/58	4.29	41.59	9.83	33.98	57.21
Huarmeyano × NASPOT 7	4.31	41.86	11.01	33.49	57.47
Huarmeyano × SPK004	4.26	41.62	9.88	34.53	56.37
Huarmeyano × NASPOT 10 O	4.30	41.51	11.34	34.58	57.05
Huarmeyano × NK259L	4.23	41.53	10.88	33.63	56.50

1 Results from the no-choice bioassay trial.

**Figure 2:**
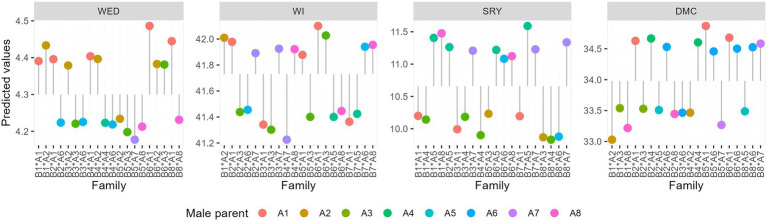
Best and worst performing families based on best linear unbiased predictors (BLUPs) for weevil severity, weevil incidence, storage root yield, and dry matter content across four environments.

Overall SCA values across families for weevil severity and incidence, storage root yield, and dry matter content were 4.3, 41.7%, 10.7 t ha^−1^, and 34%, respectively. SCA predictions for WED in the 64 families varied from 4.18 to 4.5 (Table 7; Figure 2). Families that displayed desirable SCA effects for WED by exhibiting the lowest levels of severity included “Mugande” × NASPOT 10 O (B5 × A7), and “Mugande” × “Dimbuka-bukulula” (B5 x A3) whereas NASPOT 11 × “Ejumula” (B6 × A1), “Huarmeyano” × “Ejumula” (B8 x A1), and “Resisto” × NASPOT 1 (B1 ×A2) recorded the highest levels (Figure 2).

Specific combining ability (SCA) prediction for sweetpotato weevil incidence for families ranged from 41.2 to 42.1%. Families that exhibited superior SCA for WI by displaying lowest mean percentages included “Wagabolige” × NASPOT 10 O (B4 × A7) and NASPOT 5 × “Dimbuka-bukulula” (B3 × A3) whereas NASPOT 11 × “Ejumula” (B6 × A1) and NASPOT 11 × “Dimbuka-bukulula” (B6 × A3) recorded the highest levels.

Considering SRYs, SCA effects for the 64 families ranged from 9.83 to 11.59 t ha^−1^ (Table 7). The families, “New Kawogo” × NASPOT 7 (B7 × A5), and “Resisto” × NK259L (B1 × A3) recorded the highest while “Huarmeyano” × NASPOT5/58 (B8 × A4) and “Huarmeyano” × “Dimbuka-bukulula” (B8 × A3) had the lowest means (Figure 2). SCA predictions for dry matter contents for families ranged from 30.0 to 34.8% (Table 7). The highest values were observed in “Mugande” × “Ejumula” (B5 × A1) and NASPOT 11 x “Ejumula” (B6 × A1), whereas the lowest were recorded in “Resisto” × NASPOT 1 (B1 × A2) and “Resisto” × NK259L (B1 × A8; Figure 2).

In the no-choice feeding bioassay, predicted means for the number of adult weevils in families ranged from 55 to 59. The families “Wagabolige” × NASPOT 5/58 (B4 × A4) and NASPOT 11 × NASPOT 10 O (B6 × A7) recorded the highest numbers of mean adult weevils whereas “Magabali” × NK259L (B2 × A8) and Magabali × NASPOT 7 (B2 × A5) had the lowest SCA effects for CP. It was observed that the cross “NASPOT 11” x “Ejumula” had the lowest SCA effects for weevil severity and weevil incidence and as such, displayed the highest levels of susceptibility to sweetpotato weevils among the 64 families studied. Overall, the top five crosses in terms of sweetpotato weevil resistance were; “Wagabolige” × NASPOT 10 O, NASPOT 5 × “Dimbuka-bukulula,” “Mugande” × “Dimbuka-bukulula,” and NASPOT 11 × NASPOT 7.

## Discussion

### Performance of progenies across environments

This study was aimed at identifying the best performing parental genotypes and crosses with reference to weevil (*Cylas* spp.) resistance, storage root yield, and dry matter content in the MDP sweetpotato population. The highly significant variance components for weevil severity and incidence observed in our trials highlight the possibility of selecting genotypes and families from within this population that have more resistance to *Cylas* spp. and perform better in terms of other key attributes such as SRY and DMC. The significant differences observed in the genotype by environment interactions for field related traits indicate that the various genotypes tested performed differently in all the study environments. This highlights the need for testing clones across environments in order to select those that suit particular production areas or can be broadly recommended. This occurrence is not new in sweetpotato, as similar observations were made by previous authors such as Gurmu et al. (2018) and Osiru et al. (2009).

The variations in mean weevil severity and incidence in genotypes within families could be attributed to the differences in their individual responses to these pests and differences in resistance mechanisms employed by the test clones. There is already evidence that the Ugandan landrace ‘New Kawogo’ is moderately resistant to *Cylas* spp. (Mwanga et al., 2001; Muyinza et al., 2012; Anyanga et al., 2013). This resistance has been ascribed to chemical compounds in the storage root latex, specifically the presence of higher concentrations of HCA esters on the root surfaces, epidermal and peridermal tissues of this variety (Stevenson et al., 2009; Anyanga et al., 2013). These esters have toxic effects on juvenile *Cylas* spp. and have a repellent effect on adult *Cylas* spp. (Anyanga et al., 2013).

Other plant attributes are also known to influence weevil population build-up in sweetpotato fields, they include: rooting depth, time taken before harvest (length of the growing season), vine thickness, root latex, root density, dry matter, and starch content (Mwanga et al., 2001; Stathers et al., 2003; Sorensen, 2009). More still, weevil populations are known to increase with higher temperature, soil cracks, and exposed storage roots during dry seasons (Jansson et al., 1987; Grüneberg et al., 2015).

The weak, negative correlations detected between weevil-related field traits and the number of adult weevils recorded in the bioassay could probably be due to the no-choice situation that was presented to the SPWs in the laboratory, given that only one clone root was placed in each jar. Usually in field situations, *Cylas* spp. have a choice and would rather feed on roots that meet their taste or that are more accessible to them for instance in terms of rooting depth (Stathers et al., 2003). However, when presented with no-choice situations such as the case in the current study, they may be forced to feed on whatever roots are available to them to ensure their survival. More still, the feeding habits of *Cylas* spp. are somehow related to the different resistance mechanisms employed by the various clones. For instance, clones such as MDP1140a (family NASPOT 11 × NASPOT 7), MDP3 (“Resisto” × “Ejumula”), and MDP1355a (“New Kawogo” × NASPOT 10 O) performed fairly well in the field, but then exhibited high infestations of weevils and high numbers of feeding holes in the no-choice feeding bioassay (Supplementary File S1). This could be attributed to non-preference (antixenosis) in the field due to factors such as deep roots, heavy pubescence, and high vine vigor that made the roots less accessible for weevils. Weevils are known to access storage roots through cracks in the soil, which normally increase in dry seasons. Varieties that are deep rooting, produce thinner roots, have heavy pubescence or more vines that cover the surface-shielding it from the sunshine, usually suffer less weevil infestation (Talekar, 1992; Stathers et al., 2003). These same clones when tested in the no-choice bioassay showed susceptibility as evidenced by the high number of adult weevils and feeding holes probably because they either lacked or had minimal HCA esters that are known to limit weevil proliferation (Anyanga et al., 2013). On the other hand, genotypes such as MDP1536f (“Huarmeyano” × SPK004) and MDP1018 (“Mugande” × NK259L) generally performed well with lower numbers of adult weevils and feeding holes in the laboratory bioassay and low weevil incidence and damage in the field. In their case, resistance could probably be by way of antibiosis as a result of the HCA esters that either repelled or led to the death of *C. puncticollis*, thereby minimizing damage. Yada et al. (2017b) identified clones that consistently displayed high levels of both field and HCA ester-based SPW resistance. Given that they appear to target different resistance mechanisms, field and laboratory-based bioassays may both be crucial in creating robust resistance to weevils. However, field screening should be prioritized because it best captures the actual conditions under which farmers grow sweetpotatoes. Laboratory phenotyping of clones for weevil resistance would otherwise be best when studying a smaller population of clones as this would ensure more efficiency in data collection. We suggest that more studies be designed to better understand the biology of *Cylas* spp. for more targeted control.

The relatively low mean storage root yields of sweetpotato observed in this study were mainly due to the poor performance of the trial at Abi ZARDI (season 2019B). This trial was planted towards the end of the rains and as a result, minimal rainfall was received during the growing period (330 mm). As a result, the lowest storage root yieldsand highest dry matter contents were recorded during that season. Shumbusha et al. (2014) noted that variations in DMC of sweetpotato roots could be attributed to weather patterns, soil types, and pest and diseases. The higher levels of weevil severity and incidence recorded at Abi ZARDI compared to Ngetta ZARDI may be due to the fact that Abi is located in an area that is generally hotter and drier (with less annual rainfall) than Ngetta (Table 2). Drought stress is known to negatively affect the field establishment of sweetpotatoes, which lowers their resilience to attack by *Cylas* spp., thereby escalating weevil damage (Stathers et al., 2003; Anyanga et al., 2017). This could explain why most genotypes performed better in Ngetta compared to Abi in terms of weevil severity and incidence.

### General and specific combining ability

Variance components for GCA for female and GCAf by environment interaction were important for WI and WED, respectively, and even more for SRY and DMC. The significant GCA and SCA variances observed for the traits in the field study indicate that both additive and non-additive effects are important in the expression of these traits. This indicates that both clonal selection and hybridization could be successfully employed in breeding depending on the trait (Acquaah, 2007). In our study, weevil-related traits did not show important SCA, though they were significant for SRY and DMC. Previous authors reported similar findings for weevil damage, storage root yield, and dry matter content while studying other sweetpotato populations. Kagimbo et al. (2019) reported that both additive and non-additive gene effects were important in controlling sweetpotato weevil resistance, although additive effects played a greater role. The case is similar for storage root yield and dry matter content (Rukundo et al., 2017; Gurmu et al., 2018; Kagimbo et al., 2019). Fasahat et al. (2016) observed that generally, both additive and non-additive gene action are important in governing pest resistance in many crops of commercial interest. The non-significant variance components observed for males, families, and their interactions with environments in terms of weevil severity and incidence, indicate that GCA for males and SCA generally performed in a similar manner across environments for these traits hence, did not contribute much to distinguishing best performing males and families. Similar observations were made by Kagimbo et al. (2019), who studied a population of 36 sweetpotato families in two locations in Tanzania and reported non-significant family-by-environment interactions.

Parental genotypes that were the best in terms of weevil resistance included “Mugande,” NASPOT 5, “Dimbuka-bukulula,” and “Wagabolige” whereas “New kawogo” and NASPOT 11 were the best combiners for SRY and DMC. The families that displayed highest levels of weevil resistance included; “Wagabolige” × NASPOT 10 O, NASPOT 5 × “Dimbuka-bukulula,” “Mugande” × “Dimbuka-bukulula,” and NASPOT 11 × NASPOT 7. On the other hand, the families “New kawogo” × NASPOT 7 and “Mugande” × “Ejumula” performed best in terms of SRY and DMC, respectively, (Figure 2). Parental genotypes that display high GCA values for specific traits of interest are usually the most suited for integration into breeding programs considering that there would be more transmission of those traits to their offspring because of the additive effects. Crosses with higher SCA values, however, are specifically preferred in the development of hybrid programs (De la Cruz-Lázaro et al., 2010; Ferrari et al., 2018). It is therefore recommended that the best performing parental genotypes and families identified through this study be deployed in view of this.

### Heritability estimates

Heritability is known to influence the genetic improvement of traits, with a higher heritability usually associated with fewer genes controlling the given trait, making trait improvement relatively easier using conventional breeding methods (Ma Teresa et al., 1994; Courtney et al., 2008; Mwije et al., 2014). The narrow sense heritability ( $h^{2}$ ) estimates for weevil resistance (0.35), storage root yield (0.36), and dry matter content (0.45) obtained in the present study are considered moderate. This suggests that selection for these traits might be somewhat challenging, although it could be done with a strong emphasis on using the best offspring as the parental genotypes for future hybridization. The narrow sense heritability estimate for weevil resistance obtained in this study, is similar to that reported by Thompson et al. (1994) who estimated $h^{2}$ of 0.35 for the same trait through parent-offspring regression with data obtained from root weevil damage. The $h^{2}$ estimates reported in the current study were based on a model that included pedigree information and consequently, the genetic variance was purely additive. However, the weevil resistance $h^{2}$ estimate somewhat contrasted with what was reported by some previous researchers. For instance, Kagimbo et al. (2019) and Odama (2021) reported 0.77 and 0.19, respectively, as $h^{2}$ for weevil damage. The narrow sense heritability for weevil resistance based on weevil incidence was <0.01 due to the existence of large residuals that dropped the value close to zero. However, previous authors including Kagimbo et al. (2019) and Thompson et al. (1994) reported 0.78 and 0.52 as narrow sense heritability for this particular trait. For the case of storage root yield and dry matter content, our $h^{2}\;$ estimates are higher than those reported by Gurmu et al. (2018) who obtained 0.20 and 0.19, respectively, for the two traits, and Odama (2021) who reported 0.28 and 0.32, respectively, for SRY and DMC.

Heritability estimates are known to vary with different populations, test environments, and methods of computation and can also be affected by bias and poor statistical precision (Acquaah, 2007; Agbahoungba et al., 2018). In the case of sweetpotato, the differences could also be attributed to the high levels of heterozygosity and the hexaploid nature of the crop (Gurmu et al., 2018). Falconer and Mackay (1996) noted that heritability cannot be fully exploited in heterozygotes because of the presence of non-additive gene action. Gasura et al. (2008) further indicated that the use of marker assisted selection in breeding for traits with low heritability could lead to enhanced selection efficiency. Considering the low to moderate levels of narrow sense heritability estimates reported in this study, it may be best to implement genomic assisted selection alongside conventional breeding for key traits in sweetpotato. This could not only enhance but also quicken research outputs in breeding for weevil resistance and other key sweetpotato traits.

## Conclusion

In the present study, both additive and non-additive effects were found to be significant in the expression of weevil resistance, storage root yield, and dry matter content as revealed by the significant GCA and SCA estimates. This, coupled with the moderate levels of narrow-sense heritabilities estimated, suggests that these traits could be enhanced through conventional breeding *via* hybridization and progeny selection. However, selection for weevil resistance and storage root yield may be challenging for breeders. Nonetheless, future effort in breeding for these traits could potentially be boosted through applying genomics-assisted breeding.

Parental genotypes that proved to be the best general combiners for weevil resistance included NASPOT 5, “Mugande,” “Wagabolige,” and “Dimbuka-bukulula” whereas “New Kawogo” and NASPOT 11 were superior for storage root yield and dry matter content. Based on SCA effects, the families “Wagabolige” × NASPOT 10 O, NASPOT 5 × “Dimbuka-bukulula,” “Mugande” × “Dimbuka-bukulula,” and NASPOT 11 × NASPOT 7 displayed the highest resistance to *Cylas* spp., whereas “New kawogo” × NASPOT 7 and “Mugande” x “Ejumula” were the best in terms of storage root yield and dry matter content, respectively. We recommend the superior parents and families identified through this study for further research involving the genetic improvement of these traits.

## Data availability statement

The original contributions presented in the study are included in the article/Supplementary material, further inquiries can be directed to the corresponding author.

## Author contributions

IM was involved in data collection, analysis, and writing the manuscript. JK, BY, HT, MO-S, RE, and PG were involved in supervision. PM, RO, AA, DC, MA, and BMO took part in data collection. GP and IGS participated in data analysis. RS, GP, HC, BAO, CY, and BY conceptualized the work and reviewed the manuscript. All authors contributed to the article and approved the submitted version.

## Funding

This research was funded by the Sweetpotato Genetic Advances and Innovative Seed Systems (SweetGAINS) project (Sub award Number: 2019-3125-01) from North Carolina State University to NaCRRI with the primary grant from the Bill and Melinda Gates Foundation (BMGF; Contract ID: OPP1213329) to the International Potato Center (CIP) (Contract ID: OPP1213329). The research was also funded through the BMGF Program for Emerging Agricultural Research Leaders (PEARL I) project, “Biochemistry-based Selection and Development of Nutrient Rich, Weevil Resistant Sweetpotato Varieties in Uganda” (Contract ID: OPP1112515) and the Genomic Tools for Sweetpotato Improvement (GT4SP) project (Contract ID: OPP1052983). Makerere University Regional Center for Crop Improvement (MaRCCI), an African Centre of Excellence (ACE) for plant breeding training supported by the World Bank (Grant ID Credit No. 57970 UG), provided additional financial support for research and full payment for graduate training expenses.

## Conflict of interest

The authors declare that the research was conducted in the absence of any commercial or financial relationships that could be construed as a potential conflict of interest.

## Publisher’s note

All claims expressed in this article are solely those of the authors and do not necessarily represent those of their affiliated organizations, or those of the publisher, the editors and the reviewers. Any product that may be evaluated in this article, or claim that may be made by its manufacturer, is not guaranteed or endorsed by the publisher.
